# Fighting Fat With Fat: n-3 Polyunsaturated Fatty Acids and Adipose Deposition in Broiler Chickens

**DOI:** 10.3389/fphys.2021.755317

**Published:** 2021-09-29

**Authors:** Minjeong Kim, Brynn H. Voy

**Affiliations:** Department of Animal Science, The University of Tennessee, Knoxville, Knoxville, TN, United States

**Keywords:** adipose development, adiposity, broiler chickens, DHA, EPA, hen diet, omega-3 polyunsaturated fatty acid

## Abstract

Modern broiler chickens are incredibly efficient, but they accumulate more adipose tissue than is physiologically necessary due to inadvertent consequences of selection for rapid growth. Accumulation of excess adipose tissue wastes feed in birds raised for market, and it compromises well-being in broiler-breeders. Studies driven by the obesity epidemic in humans demonstrate that the fatty acid profile of the diet influences adipose tissue growth and metabolism in ways that can be manipulated to reduce fat accretion. Omega-3 polyunsaturated fatty acids (n-3 PUFA) can inhibit adipocyte differentiation, induce fatty acid oxidation, and enhance energy expenditure, all of which can counteract the accretion of excess adipose tissue. This mini-review summarizes efforts to counteract the tendency for fat accretion in broilers by enriching the diet in n-3 PUFA.

## Introduction

Poultry meat is a major source of protein for much of the world. Due to population growth, rising incomes, and concerns about both health and sustainability, global demand is expected to continue to grow around the globe. Current projections are that poultry will account for 41% of all protein consumption from meat by 2030 (OECD and FAO, 2021). Broiler chickens are produced for meat, and genetic selection has produced remarkable advances in growth of broilers over the past several decades. For example, a 35 days broiler weighed approximately 1.4 kg in 1985 and more than 2.4 kg in 2010, largely due to increase in muscle mass (Siegel, 2014). Inadvertently, selection of broilers for rapid growth also increased their tendency to accumulate excess adipose tissue (Zuidhof et al., 2014). Adipose tissue in poultry is not commercially valuable, and it effectively wastes feed by allocating it away from muscle. Physiologically, the need for adipose tissue as an energy reservoir is minimal in market birds that have almost constant access to feed. In broiler-breeders, excess fat accretion also compromises fertility and welfare. The tendency to deposit excess adipose has in part been suppressed in commercial lines of broilers through refined approaches to selection (Siegel, 2014; Zuidhof et al., 2014). However, even a modest misallocation of feed, the most expensive component of production, significantly increases costs given the scale of commercial broiler production. The default approach to preventing fat accretion is feed restriction, but this has a negative impact on weight gain and meat yield and is also a welfare concern (Jackson et al., 1982; Lindholm et al., 2018).

### Adipose Tissue Basics in Broilers

Chickens store energy as lipids in subcutaneous depots found near the legs and neck, and in a visceral depot in the abdomen. Subcutaneous fat is the first depot to develop and becomes visible in the embryo by embryonic day 12 (E12) (Speake et al., 1993). Adipocytes develop from a mesenchymal stem cell pool that gives rise to preadipocytes, a cell type that is committed to an adipocyte fate. Preadipocytes then differentiate and acquire a mature, lipid-storing adipocyte phenotype through a series of orchestrated changes in gene expression that are similar to those described for mammals (Rosen and MacDougald, 2006). In the embryo, development of subcutaneous fat coincides with a sharp increase in extraction of fatty acids from the yolk (Speake et al., 1998). Subcutaneous adipocytes grow rapidly as these fatty acids are taken up and stored as triacylglycerol until E19, when they are mobilized by lipolysis to provide the energy needed at hatch (Speake et al., 1993). Abdominal fat, which becomes the main site of triglyceride storage in the mature bird, develops after hatch and becomes clearly visible around 7 days of age. Adipose depots grow rapidly in broiler chicks through increases in adipocyte number (hyperplasia), as preadipocytes proliferate and undergo differentiation, and size (hypertrophy) as mature fat cells take up and store fatty acids (Bai et al., 2015). Both processes are very active during the first several weeks of life, after which hypertrophy becomes the dominant mechanism of fat deposition (Cartwright, 1991). Fatty acids that are taken up from the circulation and stored in broiler adipocytes come primarily from the liver, the main site of *de novo* lipogenesis in avians (Leveille et al., 1975). Fatty acids synthesized by the liver from glucose and acetate are packaged into VLDL molecules, which ferry them to adipocytes for uptake and storage. Hepatic synthesis of lipids plays a major role in adipose deposition in broilers. Divergent selection on serum VLDL level has been used to produce lines of broilers that differ significantly in fatness, illustrating the key role of the liver (Leclercq et al., 1980; Whitehead and Griffin, 1984; Baéza and Le Bihan-Duval, 2013; Resnyk et al., 2013). In addition, intrinsic differences in adipose development also contribute to excessive fat accretion in broilers. For example, significant increases in both adipocyte number and size appear as early as 2 weeks of age in broiler lines that have higher fat mass as they mature (Hermier et al., 1989).

### Dietary Fatty Acids and Adipose Growth

Paradoxically, certain types of dietary fatty acids have been shown to inhibit accumulation of adipose tissue in a variety of species. Polyunsaturated fatty acids (PUFAs) are classified as n-3 or n-6 based on the position of the first double bond in relation to the methyl end of the molecule. Linoleic acid (LA; 18:2 n-6) and alpha linolenic acid (ALA; 18:3 n-3) are the main dietary n-6 and n-3 fatty acids, respectively. These fatty acids are required for the formation of cell membranes but cannot be synthesized endogenously and therefore must be provided in the diet (Smith and Mukhopadhyay, 2012). Linoleic acid is enriched in a variety of common plant oils, such as those derived from corn, soybean, and safflower. Alpha linolenic acid, which is less abundant than LA, is usually obtained from flaxseed, rapeseed, and walnuts. When taken into the cell, ALA and LA can be used in formation of cell membranes, oxidized for energy, or used to synthesize longer and more highly unsaturated PUFA through a series of sequential elongation and desaturation steps (Jakobsson et al., 2006). Some of these longer chain species have unique bioactive properties through their ability to bind and activate lipid-responsive transcription factors, such as members of the PPAR family of nuclear receptors. In addition, the 20 carbon products of LA (arachidonic acid; ARA, 20:4 n-6) and ALA (eicosapentaenoic acid, EPA; 20:5 n-3) are used as substrates to produce eicosanoids, a broad family of lipid mediators that interact with various cell signaling pathways. The seemingly modest structural difference between LA and ALA has marked effects on the biochemical properties of these fatty acids and their metabolites. For example, eicosanoids produced from ARA are primarily proinflammatory, while those synthesized from EPA tend to be anti-inflammatory (Calder, 2017). The same enzymes catalyze elongation and desaturation of n-3 and n-6 PUFA. As a result, the relative synthesis of longer chain n-3 and n-6 species is determined by the ratio of n-3/n-6 in the diet.

Extensive *in vivo* and *in vitro* studies indicate that n-3 and n-6 PUFA differentially regulate adiposity in multiple species. In particular, diets enriched with n-3 PUFA are associated with reduced fat accretion (Rokling-Andersen et al., 2009; Torres-Castillo et al., 2018; Ballester et al., 2020; Chen et al., 2020; Riera-Heredia et al., 2020). At the cellular level, n-3 PUFA have been shown to suppress adipogenesis, attenuate lipid accumulation in adipocytes, and promote an oxidative adipocyte phenotype (Kim et al., 2006; Manickam et al., 2010; An et al., 2012; Fleckenstein-Elsen et al., 2016). Conversely, n-6 PUFAs tend to be pro-adipogenic (Gaillard et al., 1989; Negrel et al., 1989; Massiera et al., 2003). The anti-obesogenic effects of n-3 PUFA are most strongly associated with actions of EPA and docosahexaenoic acid (DHA, 22:6 n-3), the characteristic fatty acids of marine oils (Flachs et al., 2009). These two fatty acids, which are synthesized in algae and consumed by fish, influence a range of pathways that regulate adipose growth and adipocyte metabolism. Each fatty acid can bind and activate PPARA, a nuclear receptor that transcriptionally increases fatty acid oxidation (Diep et al., 2000; Zúñiga et al., 2011). In addition, EPA and DHA activate signaling through AMPK and promote mitochondrial biogenesis, both of which augment fatty acid oxidation (Siriwardhana et al., 2012). They also increase synthesis of the insulin-sensitizing adipokine adiponectin, which further activates AMPK and amplifies energy expenditure in adipocytes (Itoh et al., 2007; Song et al., 2017). EPA and DHA are also used to synthesize several classes of lipid mediators, including eicosanoids and resolvins as well as other novel metabolites that suppress inflammation and limit metabolic stress in adipose tissue (Kuda et al., 2018). These actions indirectly oppose fat accretion by preventing inflammatory cascades that disrupt the efficient metabolism of glucose and fatty acids in adipocytes. Finally, EPA and DHA promote the browning of white adipocytes, which further enhances oxidative metabolism in adipose tissue and prevents fat accretion (Fleckenstein-Elsen et al., 2016). Browning is the process by which white adipocytes acquire the highly oxidative phenotype associated with mitochondria-rich brown fat cells. Browning includes induction of uncoupling protein 1 (UCP1), the characteristic protein of brown adipocytes, which uncouples mitochondrial respiration from ATP synthesis. These actions of EPA and DHA may not be relevant to broilers, as they are thought to lack brown adipocytes due to loss of the UCP1 gene from avian genomes (Mezentseva et al., 2008). However, a recent study demonstrates the existence of brown-like fat cells in broiler chicks (Sotome et al., 2021). Collectively, ample evidence from multiple species suggests that dietary n-3 PUFA, particularly EPA and DHA, may be a tool for reducing fat accretion in broilers.

### Reducing Fat Accretion in Broilers With n-3 Polyunsaturated Fatty Acids

Broiler diets are typically formulated with 2–5% added fat to support the intense energetic demands of rapid growth. Fat is often supplied using corn or soybean oil, both of which are readily available, cost-effective and enriched in LA. Combined with the use of corn and soybean meals as the diet base, this results in commercial diets that are heavily skewed toward high n-6/n-3 PUFA. In this context, commercial broiler diets are comparable to the modern human diet, in which high n-6/n-3 ratios have been associated with increased prevalence of obesity (Chilton et al., 2017). Considering the beneficial effects on fat accretion in other species, several groups have tested the ability to limit fat accretion in broilers by replacing a portion of LA-rich oils or saturated fat in the diet with sources enriched in n-3 PUFA. These have used a variety of experimental designs, including variation in age at which experimental diets were introduced, different amounts of added fat, and varying duration of feeding (Table 1). Nonetheless, they provide evidence that n-3 PUFA are also a promising tool to reduce fat accretion in broilers.

**TABLE 1:** Studies that investigated different dietary sources of n-3 PUFA and fat deposition in broiler chickens.

References	n-3 PUFA source	Experimental diet	Animal (age)	Feeding period	Relevant phenotypes measured	Significant effects
Crespo and Esteve-Garcia, 2001	Linseed oil	(6 or 10%) • Tallow (T) • Olive oil (OO) • Sunflower oil (SO) • Linseed oil (LO)	Broiler chickens (21 days)	22 days/29 days	• Growth performance • Abdominal fat pad	•↑ feed efficiency •↓ abdominal fat
Crespo and Esteve-Garcia, 2002	Linseed oil	(10%) • Tallow (SFA) • Sunflower oil (SO) • Linseed oil (LO)	Broiler chickens (27 days)	27 days	• Abdominal fat mass	•↓ abdominal and body fat deposition in LO compared with SFA or SO
Ferrini et al., 2008	Linseed oil	(10%) • Tallow (T) • Sunflower oil rich in oleic acid (SOO) • SO rich in linoleic acid (SOL) • Linseed oil (LO) • Mix of fats (M: 55% T + 35% LO + 10% SOL)	Female broiler chickens (1 day)	42 days	• Abdominal fat mass	•↓ abdominal fat% (reduced by 30%) in LO compared to saturated diet
Ferrini et al., 2010	Linseed oil	(10%) • Basal diet (BS) containing 0.5% sunflower oil • Tallow diet (TA) • Linseed diet (LO)	Female broiler chickens (1 day)	36 days	• Growth performance • Abdominal fat mass	•↑ BWG •↓ abdominal fat deposition
González-Ortiz et al., 2013	FO and LO mixture	(10%) • Tallow diet (S) • A blend of fish oil and linseed oil (N3 diet)	Female broiler chickens (14 days)	37 days	• Abdominal fat mass • Adipocyte size	•↓ abdominal fat% •↓ adipocyte size
Torchon et al., 2017	Fish oil Flaxseed oil	(8%) • Lard (saturated) • Fish oil (FO) • Flaxseed oil • Canola oil	Broiler chickens (7 days)	24 days	• Growth performance • Abdominal fat mass • Adipocyte size	•↓ adipocyte size •↑ plasma non-esterified fatty acid levels (lipolysis)
Ibrahim et al., 2018	Fish oil Linseed oil	(4–4.5%) • Sunflower (C) • Fish oil (FO) • C1:FO1 • C3:FO1 • Linseed oil (LO) • C1:LO1 • C3:LO1	Broiler chickens (1 day)	42 days	• Growth performance • Abdominal fat mass	•↑ BW, BWG/↓ FCR •↓ abdominal fat% •↓ TG and total cholesterol/↑ HDL-C/↓ LDL-C and VLDL
Long et al., 2018	Microalgae (MA)	(3%) • Soybean oil (SO) • MA 1: SO 2 • MA 2: SO 1	Male broiler chickens (1 day)	42 days	• Growth performance • Abdominal fat mass	•↑ BWG/↓ FCR •↑ liver %, ↓ abdominal fat % •↑ serum glucose/↓ cholesterol •↑ superoxide dismutase, ↑ antioxidant capacity

Fish and algal oils are the most direct way to increase dietary delivery of EPA and DHA to broilers. However, they are relatively expensive and more prone to lipid peroxidation than sources of ALA. Newman et al. (2002) replaced tallow (a source of saturated fat) with either fish oil or sunflower oil (as an n-6 PUFA control) in the diets of broiler chicks beginning at 3 weeks of age. After 5 weeks of feeding, fish oil significantly reduced abdominal fat pad weight and increased the breast muscle: abdominal fat ratio in comparison to tallow controls. However, comparable effects were seen with the sunflower oil diet (Newman et al., 2002). Unlike most vertebrates, many types of algae can efficiently synthesize long-chain n-3 PUFA and are thus rich sources of EPA and DHA. Microalgae are microscopic algae species of commercial interest as renewable sources of biofuel production and animal feeds (Khan et al., 2018). Long et al. (2018) used a source of dehydrated microalgal biomass to efficiently deliver DHA in broiler diets. The biomass consisted of 64% fat made up primarily of palmitate (60%) and DHA (29%). Microalgae were added to the diet and 1 and 2% by weight, with the balance of fat (up to 3% total) provided by soybean oil, and chicks were fed from 1 to 42 days. Both the 1 and 2% microalgae diets significantly reduced adiposity at 42 days. In addition, both diets significantly improved feed efficiency and increased weight gain, and improved antioxidant status, resulting in broader effects on performance than just reduced fatness. These results demonstrate that replacing even part of an n-6 PUFA source in the broiler diet with a DHA-rich alternative has beneficial effects on fat accretion.

Flaxseed and other oils rich in ALA provide the substrate for EPA and DHA synthesis, but the efficiency of ALA conversion to longer chain n-3 PUFA is thought to be very low in vertebrates due to catalytic properties of the elongase enzymes in the PUFA synthesis pathway (Arterburn et al., 2006; Brenna et al., 2009; Gregory et al., 2011). However, the chicken elongase enzymes (Elongation of Very Long Chain Fatty Acids-Like 2 and 5 (ELOVL2 and ELOVL5) in this pathway have enhanced ability to synthesize DHA from ALA (Zuidhof et al., 2009; Gregory et al., 2013). ELOVL5 is highly expressed in broiler adipose tissue, including in the embryo (Mihelic et al., 2020). Unlike in most mammals, supplementing broiler diets with ALA significantly enriches tissues in EPA and DHA (Poureslami et al., 2010; Kartikasari et al., 2012). Accordingly, several studies have been reported that linseed oil inclusion in broiler diet suppressed abdominal fat deposition and enhanced growth performance compared with saturated fat sources, such as tallow (Crespo and Esteve-Garcia, 2001, 2002; Ferrini et al., 2010; González-Ortiz et al., 2013) or n-6 PUFA sources such as sunflower oil (Crespo and Esteve-Garcia, 2002; Ibrahim et al., 2018). For example, supplementing broiler diets with flaxseed oil (10%) reduced abdominal fat by 30% compared to equivalent amounts of saturated or monounsaturated fatty acids (Ferrini et al., 2008). Both fish and flaxseed oils have also been shown to reduce adipocyte size in broiler chicks, suggesting that they act relatively early on adipose development (Torchon et al., 2017). The relative effects of fish oil and flaxseed oil as replacements for sunflower oil have also been compared. Broilers were fed diets in which all or part of sunflower oil (4%) was replaced by either flaxseed or fish oil (Ibrahim et al., 2018). Each diet significantly reduced abdominal fat percentage compared to sunflower controls, while feed efficiency, gain, and antioxidant status were significantly improved. The experimental diets also improved muscle tissue weight (breast and thigh) and decreased breast intramuscular fat (IMF) content. The diets that replaced sunflower with flaxseed oil had very low levels of EPA and DHA, as did the sunflower control. However, fatty acid analyses of breast muscle further support the ability for chickens to efficiently synthesize these fatty acids from ALA. Tissue levels of EPA and DHA were significantly increased in all experimental diets, except for the lowest level (5:1, sunflower: flaxseed) of ALA inclusion.

### Potential to Reduce Fat Accretion Through n-3 Polyunsaturated Fatty Acids in the Hen Diet

Given the scale of broiler production, replacing standard fat sources with more expensive ones, such as flaxseed or fish oil, would impose a significant cost to the industry. Developmental programming through the diet of the broiler-breeder hen is a potential alternative means to reduce fat accretion in broilers. Developmental programming refers to the ability of embryonic exposures to exert persistent effects on the physiology of offspring after birth. This concept forms the basis of the Barker hypothesis, which was proposed in 1992 to explain the influence of factors during fetal development on the risk of diseases as adults (Barker, 1992). Programming occurs in part through epigenetic mechanisms, in which pre- and perinatal factors modify the genome through methylation, chromatin acetylation, and other non-sequence-based changes. These modifications persist through cell divisions and can influence gene expression and subsequently phenotypes throughout the life of the organism as well as across multiple generations. The concept of developmental programming has been applied to a plethora of embryonic exposures, but it is rooted in the impact of the maternal diet on offspring (Barker and Osmond, 1986). Studies in mammals indicate that energy metabolism and adiposity are especially sensitive to developmental programming by the maternal diet (Holness et al., 2000; George et al., 2012; Du et al., 2013; Lukaszewski et al., 2013; Ong and Guest, 2018). For example, energy deficit in the maternal diet during pregnancy increases adiposity in offspring after birth (Howie et al., 2012). A growing body of evidence from other species suggests that n-3 PUFA can act very early in life to influence adiposity and metabolism through developmental programming. For example, higher maternal blood levels of n-3 vs. n-6 PUFA during pregnancy have been associated with reduced body fat and increased leanness in children (Donahue et al., 2011; Vidakovic et al., 2016a, b). Experimental studies demonstrate that the relative abundance of n-6 vs. n-3 fatty acids in the perinatal period influences adipogenesis and fat accretion. Mice born from dams with low n-6/n-3 ratios due to genetic manipulation had half the body fat, increased energy expenditure and fatty acid oxidation, and more but smaller adipocytes as adults compared to mice born to wild type dams (Rudolph et al., 2018).

Avians are ideally suited for developmental programming by fatty acids due to the relationship between the hen diet and the yolk, and the role of yolk fatty acids in supporting the developing embryo. Approximately 60% of the yolk’s dry mass consists of lipids that are transported to the egg from the hen’s liver (Speake et al., 1998). This lipid supply includes triglycerides that provide the embryo with fatty acids needed for energy, and phospholipids that serve as building blocks for cell membranes. Virtually all of the triglyceride and phospholipid pools in the yolk are utilized by the time of hatch (Noble and Moore, 1964). Saturated and monounsaturated fatty acids delivered to the yolk can be synthesized in the liver through *de novo* lipogenesis from carbohydrate, but the PUFA components come directly from the diet. Therefore, the n-3 and n-6 profile of the yolk, and subsequently of the tissues of the developing embryo, is determined almost exclusively by the diet of the hen (Cherian and Sim, 1991, 1993; Cherian et al., 1997). This appears to be particularly true for n-3 PUFA, which are preferentially incorporated into phospholipids of the developing embryo (Speake et al., 1998). Fish oil is the most direct means to enrich eggs and developing embryos in EPA and DHA, as the hen liver directly incorporates these species into phospholipids that are packaged for delivery to the yolk. However, oils such as flaxseed can have similar effects because of the unique properties of PUFA synthesis enzymes in chicken (Cherian et al., 1997; Gregory et al., 2013). The hen liver elongates and desaturates a portion of dietary ALA before packaging for delivery to the yolk. During embryonic development, both the yolk sac and the tissues of the embryo convert additional ALA to EPA and DHA, amplifying the effects of the hen diet (Noble, 1987; Noble and Cocchi, 1990). Therefore, oils that are more affordable and more stable than fish oil could feasibly be used for dietary developmental programming in production. Several studies have utilized the relationship between fatty acids in the hen diet and those of the embryo to demonstrate the potential for developmental programming by n-3 PUFA in chickens. For example, enriching the hen diet in different sources of n-3 PUFA has been shown to influence immunocompetence, synthesis of inflammatory mediators, bone mineralization, and antioxidant status in offspring (Hall et al., 2007; Bautista-Ortega et al., 2009; Bullock et al., 2014; Akbari Moghaddam Kakhki et al., 2020). Readers are referred to a recent review by Thanabalan and Kiarie (2021) for more details.

The ability to developmentally program reduced fat accretion through n-3 PUFA in the hen diet has been demonstrated in broilers. Beckford et al. (2017) fed broiler-breeder hen diets with fat provided from either corn or fish oil (2.3%) for 4 weeks. Chicks hatched from both sets of hens were fed commercial starter diets after hatch, with no modification to the fat source. Those produced from hens fed fish oil had significantly less fat accretion at 7 and 14 days, with no impact on growth or lean tissue mass. Reduced fat accretion was associated with smaller but more abundant adipocytes, consistent with inhibition of late stage of adipogenesis. Transcriptomic and proteomic analyses of adipose tissue indicated that the hen fish oil diet inhibited pathways associated with triacylglycerol synthesis, adipogenesis, mobilization of stored fatty acids from lipid droplets, and fatty acid utilization, all of which likely contributed to reduced adipose mass. This study provides compelling evidence that the broiler hen diet is a potential avenue through which to reduce fat accretion in broilers through developmental programming by n-3 PUFA.

It is important to acknowledge the potential challenges that would result from increasing the relative content of LC n-3 PUFA in production diets. In addition to a higher cost, oils rich in long-chain PUFA species are more prone to oxidation due to increased chain length and numbers of double bonds in their fatty acids. Supplementation with additional antioxidants, such as vitamin E, is often used to suppress oxidation, which can impair taste and meat quality. Alternative sources of LC n-3 PUFA that are naturally enriched in antioxidants may be able to offset this concern. For example, microalgae, a viable and effective alternative to fish oil as a source of EPA and DHA, have been shown to contain antioxidants that markedly reduce lipid peroxidation in broiler tissues (Tao et al., 2018).

## Summary

Collectively, these studies demonstrate the ability to reduce the deposition of adipose tissue in broilers by enriching the diet in n-3 PUFA. In particular, the potential to limit fat accretion through developmental programming shows promise for efficiently limiting fat deposition through the diet of the hen. Strategies to continue to improve the utilization of feed for broiler production will be increasingly important as the global population continues to grow and seek affordable sources of complete protein.

## Author Contributions

MK reviewed the studies described herein, prepared the included table, drafted the initial version of the manuscript, and proofread the final version. BV conceived the overall content, edited the draft, and approved the final version. Both authors contributed to the article and approved the submitted version.

## Conflict of Interest

The authors declare that the research was conducted in the absence of any commercial or financial relationships that could be construed as a potential conflict of interest. The handling editor declared a past co-authorship with one of the author BV.

## Publisher’s Note

All claims expressed in this article are solely those of the authors and do not necessarily represent those of their affiliated organizations, or those of the publisher, the editors and the reviewers. Any product that may be evaluated in this article, or claim that may be made by its manufacturer, is not guaranteed or endorsed by the publisher.
